# Computer Vision Syndrome: An Ophthalmic Pathology of the Modern Era

**DOI:** 10.3390/medicina59020412

**Published:** 2023-02-20

**Authors:** Irina Andreea Pavel, Camelia Margareta Bogdanici, Vlad Constantin Donica, Nicoleta Anton, Bogdan Savu, Cristina Petronela Chiriac, Cristian Dan Pavel, Silvia Cristina Salavastru

**Affiliations:** 1 Department of Ophthalmology, Faculty of Medicine, University of Medicine and Pharmacy “Grigore T. Popa”, University Street, No.16, 700115 Iasi, Romania; 2 Department of Pediatrics, Faculty of Medicine, University of Medicine and Pharmacy “Grigore T. Popa”, University Street, No.16, 700115 Iasi, Romania; 3 “Saint Spiridon” Emergency Hospital, 700111 Iasi, Romania; 4 Department of Histology, Faculty of Medicine, University of Medicine and Pharmacy “Grigore T. Popa”, University Street, No.16, 700115 Iasi, Romania

**Keywords:** computer vision syndrome, digital eye strain, gadgets

## Abstract

Digital device usage has increased significantly in last decade among all age groups, both for educational and recreational purposes. Computer vision syndrome (CVS), also known as digital eye strain (DES), represents a range of ocular, musculoskeletal, and behavioral conditions caused by prolonged use of devices with digital screens. This paper reviews the principal environmental, ocular, and musculoskeletal causes for this condition. Due to the high prevalence of DES and frequent usage of digital devices, it is important that eye care practitioners be able to provide advice and management options based on quality research evidence.

## 1. Introduction

Computer vision syndrome (CVS), also known as digital eye strain (DES), represents a pathology of the modern era characterized by the presence of various ocular, musculoskeletal, and behavioral signs and symptoms produced by the prolonged use of electronic devices with a digital screen. Among the first symptoms that appear are blurred vision, conjunctival congestion, eye fatigue, visual accommodation disorders, headaches, muscle pain in the neck and back, and attention difficulties [1]. CVS represents a current health problem that has been affecting the entire population for over two decades. All people, regardless of age, are at risk of developing this pathology because the time spent in front of digital screens is constantly increasing [2]. More and more people are using computers not only at work, but also at home in their spare time. In addition, other digital screen electronic devices, such as laptops, tablets, and smartphones, are increasingly used by all generations, regardless of age [3].

This paper analyzes the most recent and significant research in this field. The current study presents up-to-date data and information on the use of visual display terminals (VDTs), characteristic signs and symptoms, and useful therapeutic strategies.

## 2. Paper Selection

**Figure 1:**
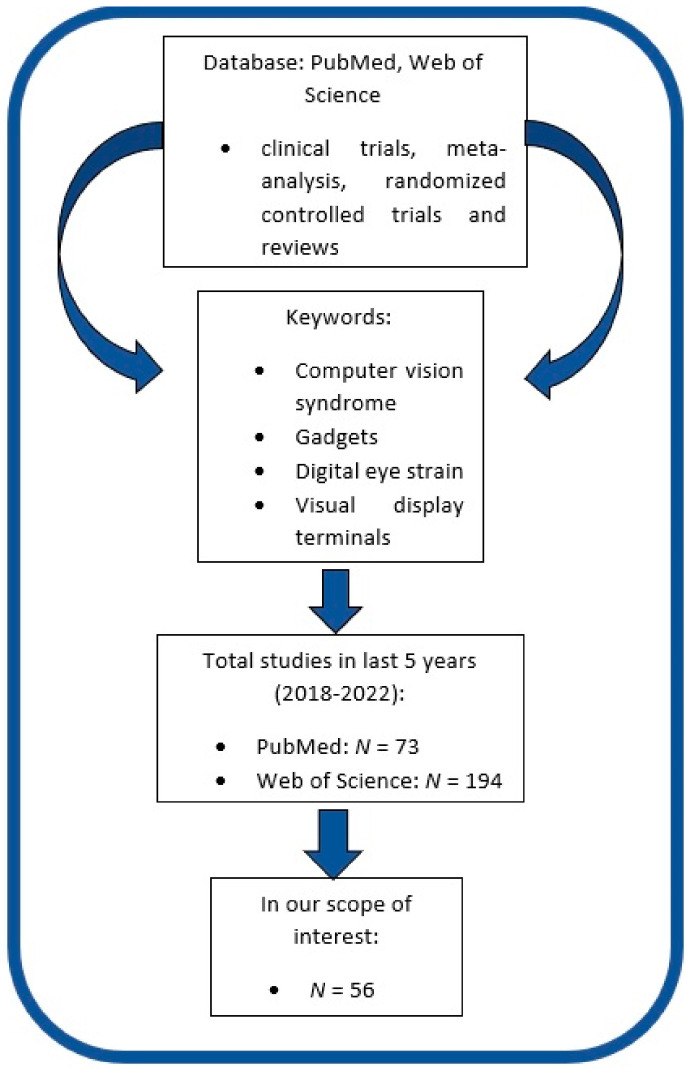
A schematic representation of the current study design.

We searched PubMed and Web of Science databases, focusing on studies published within the last 5 years. We used the following keywords: computer vision syndrome, gadgets, digital eye strain, visual display terminals, to find relevant articles on the selected topic. We compared all appropriate articles that we found from the literature and we focused on prospective and retrospective studies, metanalyses, and reviews, excluding abstracts, documents, non-English articles, editorial letters, conference posters, and preprints. Our search resulted in 267 total references, and only 56 papers were within our scope of interest. A schematic representation of the current study design is shown in Figure 1.

## 3. Asthenopia, Visual Disturbances, and Intraocular Pressure Modification

### 3.1. Asthenopia

Recent studies found that the prevalence of asthenopia among VDT users is between 55% and 81% [4].

Some studies concluded that it is still uncertain if asthenopia is associated with age during computer use [5,6], while Bhanderi et al. found that VDT use at an early age is frequently associated with asthenopia [7]. Another study identified age over 30 years old as a risk factor for VDT-related dry eyes [8].

The prevalence of asthenopia during computer use seem to be higher in females [3]. Toomingas et al. identified statistically significant association between ocular symptoms and female sex [9]. Moreover, the Osaka Study showed that dry eye disease related to prolonged computer use affect females more than males [8].

### 3.2. Accommodation and Vergence Anomalies and Amblyopia

It is quite obvious that computer operation involves prolonged near work. Vergence dysfunctions include decompensated heterophoria, poor vergence facility, and convergence insufficiency. Patients who have binocular vision difficulties experience greater ocular symptoms with extended use of the eyes [10].

Qu found that even a reduced 1 h working time with VDT can cause decreased accommodation amplitude and retraction of the near convergence point [11].

Rosenfield et al. reported no modification in vergence facility following 25 min computer work [12]. In another study conducted by the same author, it was concluded that about 20% of patients preferred an induced small exo-associated phoria compared with an ortho condition emphasizing that CVS may be enhanced by stimulating an exo-associated phoria in some patients [3].

Numerous studies found that prolonged near work during smart phone use might lead to the development of acute acquired comitant esotropia (AACE) in young adults and children [13,14,15].

Kaur et al. reported that AACE in children can be associated with acute onset diplopia which can affect their near work. If persistent for a long time, diplopia may lead to amblyopia [16].

A study showed that quality of life may be affected in young adults with amblyopia. Socio-professional orientation should be carried out as early as possible to enhance the quality of life in these patients [17].

### 3.3. Temporary Gadget-Induced Myopia

Using VDTs for a long time requires a sustained and significant accommodative effort. A recent study by Liu et al. showed that different degrees of myopia are associated depending on the type of VDT used. On the one hand, the highest myopia value was associated with prolonged use of smartphones and computers, and on the other hand, the lowest value was recorded when using tablets and watching TV [18].

### 3.4. Intraocular Pressure Changes Secondary to the Use of Digital Screens

Various studies have investigated the association between increased intraocular pressure (IOP) and smartphone use and have found important results.

Lee et al. conducted a study on 158 eyes and found that a small but significant increase in IOP was associated with viewing visual material on a smartphone [19]. Another study which was conducted on 39 participants showed that smartphone use was significantly associated with increased IOP, and the modifications of IOP were more important under the low-light condition [20].

Ha et al. investigated the effect of writing and reading on a smartphone on IOP changes in patients diagnosed with normal tension glaucoma. The authors concluded that using a smartphone in low light conditions may increase IOP in patients with known normal tension glaucoma [21].

## 4. Environmental and Work Factors

### 4.1. Surrounding Light

The various light sources surrounding the desk have a direct and major influence on ophthalmological symptoms. Light coming from a source located over a VDT diminishes the contrast of the text on VDT, leading to eye fatigue and discomfort [22].

A study conducted by Joines et al. concluded that significant benefits to visual and musculoskeletal comfort and posture have been accounted when participants used the adjustable work lights [23].

Another study conducted in four elementary and secondary schools in Sujiatun, Shenyang, China concluded that visual acuity loss could be improved in students with the help of elevated light levels which may delay the response to myopiagenic stimuli for the eyes [24].

Janosik et al. found that an illumination higher than 200 lux is suitable at the VDT workstation [25].

### 4.2. Working Hours

Toomingas et al. revealed that long working hours on VDT determine more visual symptoms and the organization of computer work should offer frequent breaks from near-work at the computer screen [9].

Using a VDT for more than 8 h a day is a risk factor for dry eyes. A work–rest schedule with breaks at every 15 min followed by micro-breaks or at every 30 min followed by 5-min breaks has been shown to significantly increase work efficiency and reduce eye and musculoskeletal discomfort [22].

A study conducted by Christensen et al. about the effect of increased smartphone screen-time showed that longer average screen-time was associated with shorter sleep duration and worse sleep-efficiency [26]. Numerous studies confirmed that excessive smartphone use affects sleep quality and has various physical and psychological side effects [27,28,29].

Auffret et al. showed in a paper published in 2022 that binocular balance can be affected by prolonged use of digital screens [30].

A study conducted on 194 participants found that older subjects and people spending more than 4 h a day using VDTs are at major risk to develop dry eye syndrome [31].

A study from 2021 showed that approximately 90% of computer users, who spend more than 3 h per day in front of the computer screen, suffer from CVS [32].

The effects of prolonged digital screen use from numerous studies on quality of life and vision are presented in Table 1 [26,27,28,29,30,31].

### 4.3. Microenvironment

Factors such as humidity less than 40% and high temperature can increase tear film evaporation, producing hyperosmolarity and eye discomfort. Furthermore, other factors that can produce ophthalmic symptoms in the indoor workplace include pollens, dust, aerosols, or chemical irritants [22].

## 5. Personal Factors

### 5.1. Ametropia

Uncorrected refractive errors can lead to increased ocular symptoms. As more and more people spend a lot of time working in front of digital screen electronic devices, it is especially important that they are able to maintain a clear image at all times throughout the use of VDTs [3].

In two similar studies, the effects of uncorrected astigmatism were analyzed while the study participants were asked to read a text from a computer screen. The authors of both studies showed that the presence of an uncorrected astigmatism of 0.50–1.00 D may cause exacerbation of ophthalmological symptoms [33,34].

The correction of presbyopia can represent a major problem for those who spend long periods of time in front of digital screens. Harris and Straker showed that laptops can be used in a variety of positions, from sitting at a desk to sitting with the laptop in the arms or even lying prone. Consequently, a spectacle correction prescribed for a desktop computer is often inadequate for a laptop [35].

### 5.2. Nicotine Use

A recent study found that smoking represents a risk factor for dry eye, cataract, age-related macular degeneration, glaucoma, and Graves’ ophthalmopathy [36].

## 6. Device-Related Factors

### 6.1. Angle of VDT

Gaze angle and viewing distance depend on the layout of the workplace, the height of the object being viewed, as well as the height of the individual.

Information provided by official authorities indicates that the center of the screen must be positioned below the horizontal level of the eye between 15 and 20 degrees. Moreover, the entire visual area of the screen must be positioned in such a way that the angle to be viewed downwards does not exceed 60 degrees. In addition, to be viewed correctly, a digital screen must be situated between 50 and 100 cm from the eye [3].

It is also important to note that smartphones can be used in the primary or downward gaze, while laptops and computers are usually used in the downward gaze [3].

These data are consistent with the results of a study which showed that participants with CVS had a much higher average viewing angle to the screen [37].

A recent study from 2021 analyzed the risk factors associated with CVS and concluded that improper viewing angle was the most common risk factor, with the edge of the screen positioned above or at the horizontal level of the eye [38].

### 6.2. Screen Resolution and Text Size

A 2021 study found the poor screen resolution, inadequate brightness of the screen, and glare that occur on older screens are risk factors increasing the severity of CVS. In addition, the small screen and font size increase eye strain and fatigue due to improper eye focus. The authors of the study concluded that the highest CVS severity was associated with inappropriate use of smartphones, while the lowest severity was associated with desktop computers [38].

Another study showed that high-resolution displays may have benefits because they present brighter and clearer images. Nevertheless, their effect on eye strain and fatigue is not well known [39].

Ranasinghe et al. found that light from the VDT can produce a glaring effect on eyes that can determine symptoms such as nearsightedness, ocular discomfort, blurred vision, and dry eye [37].

### 6.3. 3D Stereoscopic Display

Numerous studies which investigated the ocular modifications after watching a 3D display concluded that a 3D screen affects accommodation and convergence abilities, as well as tear dynamics [40,41,42].

Virtual Reality differs slightly (VR) from classic 3D displays due to the fact that it creates a full stereoscopic experience instead of a brief immersion in the 3D world. A study from 2020 showed that watching a 3D display can cause subjective symptoms such as asthenopia, motion sickness, fatigue, or head or neck pain [43].

Another study conducted in 2020 showed that binocular accommodative facilities and vergence facilities increased after 25 min of VR gaming [44].

A study conducted in 2022 analyzed the effect of Virtual Reality 3D head-mounted display (VR 3D HMD) and found that sickness was more frequent in women [45].

The results of these studies are presented in Table 2.

## 7. Ocular Surface Disorder

### 7.1. Spontaneous Blink Impairment

A study conducted in 2022 which investigated the progression of visual fatigue secondary to VDT using automatically detected blink features, concluded that blink number and mean blink duration were significantly higher and mean blink interval was significantly lower [46]. When looking at VDT for a long period of time, the tear film evaporates excessively, causing ocular discomfort [47]. To decrease the discomfort of visual fatigue, people refreshed the tear film by blinking many times and extending blink duration and, as a consequence, decreasing blink interval [46].

### 7.2. Dry Eye Disease and Tear Film Quality Prevention of Digital Screen-Induced Dry Eye

The presence of dry eye may represent a significant factor in the etiology of CVS. The environments where work spaces are placed frequently have decreased ambient humidity and forced-air heating or air conditioning which may exacerbate symptoms of dry eye [3].

A study by Akkaya et al. evaluated the side effects of long-term computer use on tear production and evaporation. The results of the study showed that long-term computer use did not influence Schirmer test values, but instead, there were significant changes in tearing break-up time (TBUT) values. The results of this study showed that long-term computer use can cause dry eye disease (DED), the evaporative type [48].

Numerous recent studies showed that prolonged digital screen use was associated with a higher risk of clinically diagnosed DED and severe symptoms of dry eye [49,50,51]. Another recent study showed that using VDT more than 8 h per day led to symptomatic dry eye compared to less than 4 h [52]. The results of these studies are presented in Table 3.

Numerous studies have investigated the association between DED and the use of digital screen electronic devices in the pediatric population. One study showed that of all gadgets, smartphones are most often associated with DED in children [53].

Another study showed that after stopping smartphone use for a month, all children experienced a decrease in symptoms caused by DED. Thus, using the smartphone with caution can increase the quality of life [54].

A recent study conducted in 2020 showed that long-term use of VDTs is associated with decreased tear film quality. Therefore, using the smartphone was associated with maintaining a lower angle of view and with a smaller extent of the exposed eyeball, and the use of the computer screen was associated with important conjunctival congestion, a lower height of the lacrimal meniscus and a higher osmolarity [55].

In order to prevent the occurrence of DED in the population using electronic devices with digital screens, various measures can be taken, such as short periods of rest of the eyes, blinking techniques, and environmental changes.

A recent study conducted in 2021 investigated the effect of blinking exercises which consisted in closing the eyes for 2 s two times and after that squeezing the eyelids together tightly for 2 s. After one month of the blinking exercises, symptoms of DED reduced, lipid layer quality improved, and TBUT increased [56].

Another strategy is called “blind working” which consists in closing the eyes when vision is not required. Compared to normal working, dry eye symptoms, ocular discomfort, and blurred vision were diminished with this method [57].

A third technique for preventing DED is the 20-20-20 rule. This method is used in order to prevent the appearance of CVS symptoms. This technique requires that every 20 min, a break is taken for 20 s, during which patients look out the window at a distance of 20 feet [58].

Regarding environmental modifications, a study found that 1 h of computer use with a desktop humidifier was associated with an enhancement in TBUT and eye comfort [59].

## 8. Extraocular Symptoms

Despite the fact that ophthalmological issues are the most frequently encountered complaint among patients with CVS, extraocular symptoms are also an important topic to research.

Previous studies have found that some people spent in front of digital screens up to 12 h daily [60,61]. Prolonged time spent in front of VDT can cause extraocular symptoms such as headaches, depression, and sleep disorders [62,63]. Musculoskeletal symptoms include pain in the neck, shoulder, or back [61,64]. Other extraocular symptoms reported in CVS are difficulties in holding objects or writing and pain in thumbs, fingers, or wrists caused by tendonitis or arthritis [65,66].

A study conducted in 2022 analyzed the ocular and musculoskeletal modifications determined by the use of digital screens in children. The research found that musculoskeletal symptoms were encountered in large numbers in all participants, which suggests that musculoskeletal changes should be analyzed and treated very seriously in CVS [67].

Headaches have been shown to be more common when the gadget is kept at a distance of less than 50 cm. For this reason, the use of smartphones makes this a significant problem [68].

Constant use of gadgets can cause an abnormal forward bending position of the neck. Neck flexion angle has been found to be greater in cases of smartphone use and may influence muscle fatigue and upper trapezius pain [69]. A recent paper from 2021 reported that the most frequent musculoskeletal symptoms were shoulder, neck, and back pain, found in approximately ½ of the participants [70].

There are other factors that cause back pain, which are represented by the location of the screens and the keyboard, as well as the positioning and model of the desk [71]. When the position of the digital screen is too high or too low, it may cause back pain and abnormal postures [72]. Uncomfortable furniture with inappropriate size and shape may also determine back pain [73].

Wrists, arms, and hands are also affected by excessive use of VDTs [74,75]. Prolonged time of using digital screen devices causes pressure on the tendons of the wrists, leading to wrist and hand pain. Carpal tunnel syndrome represents a pathology that causes numbness, pain, and tingling in the arm and hands. This condition occurs when a median nerve is pressed as it travels through the carpal tunnel in the wrist. Based on these findings, it is obvious that extended use of electronic devices can determine carpal tunnel syndrome [76].

High prevalence of dermatitis such as rosacea or seborrheic eczema, is found among digital screen users and is called “screen dermatitis”. It can produce erythema, pain, and edema [9].

## 9. Conclusions

As technology evolves, digital screen devices have become a common tool that is used for different purposes on daily basis. We found in our study that CVS represents a major public health problem, resulting in a variety of complaints and symptoms, that can dramatically affect the quality of life of the individuals.

Risk factors that lead to CVS are represented by poor screen resolution, inadequate brightness of the screen, glare, and improper viewing angle. Moreover, the small screen and font size increase eye strain and fatigue due to improper eye focus.

We found in our study that CVS is more common in children and females and the highest CVS severity is associated with inappropriate use of smartphones. In addition, 3D screens affect accommodation and convergence abilities and also tear dynamics, and smartphone usage is frequently associated with increased IOP.

Musculoskeletal symptoms associated with CVS include pain in the neck, shoulder, or back, difficulties in holding objects or writing, and pain in thumbs, fingers, or wrists. Moreover, headaches are frequently associated with smartphone use.

Preventive techniques and population education about the burden of such a lifestyle and the adequate handling of various types of gadgets must be addressed.

## Figures and Tables

**Table 1:** Published studies that analyze the effect of prolonged digital screen use on quality of life and vision.

Author and Year	Participants and Methods	Key Findings
Christensen et al., 2016 [26]	Cross-sectional analysis of 653 participants enrolled in a longitudinal cohort study conducted via the Internet, which could be accessed by any interested adult;	Long-term use of VTDs has been shown to be associated with shorter sleep times and lower sleep efficiency;
Arshad et al., 2021 [27]	Descriptive cohort study conducted on 280 students at Rawalpindi Medical University for a period of 1 month;	Excessive smartphone use affects sleep quality and has various physical and psychological side effects;
Patil et al., 2019 [28]	Questionnaire-based research conducted on 463 medical students;	Prolonged use of digital screens can affect the quality of sleep;
Grimaldi et al., 2020 [29]	Study conducted on 306 college students from various universities from Seville;	Young adults’ prolonged exposure to smartphones is significant associated with reduced physical activity, sedentary lifestyle, disturbed mood, and low sleep quality;
Auffret et al., 2022 [30]	Cross-sectional, prospective, monocentric pilot study conducted on 52 participants;	Chronic use of screens had a negative influence on binocular balance;
Rossi et al., 2019 [31]	Cross-sectional study conducted on 194 subjects; the participants were divided in two groups: VDT workers and control group;	Older participants and those who spend more than 4 h a day using VDTs are at major risk of developing dry eye syndrome;

**Table 2:** Published studies that analyze the ocular modifications after watching a 3D display.

Author and Year	Participants and Methods	Key Findings
Yum et al., 2014 [40]	A total of 38 participants were included; before and after viewing a 3D screen, the near point of accommodation, near point of convergence and the tear break-up time were analyzed;	Watching a 3D display has negative effects on accommodation, convergence and tear dynamics;
Yum et al., 2015 [41]	A total of 30 participants were included; before and after viewing a 3D screen, the near point of accommodation, near point of convergence, and the tear break-up time were analyzed;	Motion-in-depth has an important influence on ocular parameters when a 3D display is watched;
Wee et al., 2014 [42]	Conducted on 15 adults without ophthalmological pathology; before and after viewing 3D and 2D screens, accommodative capacity, ocular protection index, and total ocular symptom scores were analyzed;	Impairment of accommodative capacity and stability of the ocular surface may be causative factors of visual asthenopia in participants viewing 3D displays;
Tychsen et al., 2020 [43]	Conducted on 50 children; this study aimed to assess the safety of VR 3D headset (Virtual Reality three-dimensional binocular–stereoscopic near-eye display) in young children;	Watching a 3D display can cause subjective symptoms such as asthenopia, motion sickness, fatigue, head or neck pain;
Munsamy et al., 2020 [44]	Conducted on 62 participants with ages between 18 and 30 years; the paper investigated the modifications between accommodative and vergence facilities before and after exposure to VR device;	Binocular accommodative and vergence facilities increased after 25 min of VR gaming in emmetropic participants under 30 years of age;
Ciążyńska et al., 2022 [45]	Conducted on 45 participants; the study investigated the effects of VR 3D HMD gaming in terms of immersion, simulator sickness, breathing, and heart rates and energy expenditure during two sessions of playing on males and females;	The second game session caused sickness in both groups, more noticeably in women;

**Table 3:** Published studies that analyze the association between digital screen use and dry eye.

Author and Year	Participants and Methods	Key Findings
Hanyuda et al., 2020 [49]	Cross-sectional study which included a total of 102,582 participants aged 40–74 years;	Physical inactivity, prolonged sedentary behaviors, and use of VDT were related to increased susceptibility to DED among middle-aged to older Japanese adults;
Wang et al., 2021 [50]	Conducted on 322 participants; a questionnaire regarding lifestyle was administered, and dry eye symptomology, ocular surface characteristics, and tear film quality were evaluated;	Prolonged use of digital screens and reduced caffeine consumption were factors associated with higher chances of dry eye disease;
Wolffsohn et al., 2021 [51]	A total of 1125 participants; a demographic and lifestyle questionnaire was administered, and dry eye symptomology, ocular surface characteristics and tear film parameters were evaluated;	Risk factors associated with evaporative type DED were older age, East and South Asian ethnicity, contact lens wear, increased exposure to the digital device screen, higher psychological stress, and poorer health;
Inomata et al., 2020 [52]	Cross-sectional study including individuals in Japan who completed a questionnaire;	Extended screen exposure (>8 h per day) was positively associated with symptomatic dry eye;

## Data Availability

Data sharing is not applicable to this article.
